# The patterns of relapse and abstinence: using machine learning to identify a multidimensional signature of long-term outcome after inpatient alcohol withdrawal treatment

**DOI:** 10.3389/fpsyt.2026.1683069

**Published:** 2026-04-17

**Authors:** Florian J. Raabe, Sarah Brechtel, Charlotte Lugmair, Judith Weiser, Kolja Schiltz, Nikolaos Koutsouleris, Peter Falkai, Eva Hoch, Oliver Pogarell, Gabriele Koller, David Popovic

**Affiliations:** 1 Department of Psychiatry and Psychotherapy, Ludwig-Maximilian-University Munich (LMU) University Hospital, Ludwig-Maximilian-University Munich (LMU) Munich, Munich, Germany; 2 Max Planck Institute of Psychiatry, Munich, Germany; 3 Department of Psychiatry and Psychotherapy, Medical Center - University of Freiburg, Faculty of Medicine, University of Freiburg, Freiburg, Germany; 4 German Center for Mental Health (DZPG), Partner Site Munich-Augsburg, Munich, Germany; 5 Institute of Psychiatry, Psychology and Neuroscience, King’s College London, London, United Kingdom; 6 IFT Institut für Therapieforschung, Centre for Mental Health and Addiction Research, Munich, Germany; 7 Department of Clinical Psychology and Psychotherapy, Charlotte Fresenius University, Munich, Germany

**Keywords:** alcohol dependence, biomarkers, machine learning, outcome, relapse, withdrawal treatment

## Abstract

**Aims:**

A machine learning approach to identify a multidimensional signature associated with relapse and long-term outcome in alcohol dependence treatment.

**Design:**

In this observational naturalistic study, inpatients with alcohol dependence received qualified detoxification plus CBT (Cognitive Behavioral Therapy) and were followed up 6-months after discharge to assess abstinence and drinking behavior. Cross-validated multivariate sparse partial least squares analysis (SPLS) was used to investigate the relationship between clinical features and four long-term outcome variables.

**Setting:**

Germany.

**Participants:**

152 patients (on average 47.8 years old, 72% male) with alcohol dependence, who received inpatient qualified detoxification plus CBT.

**Measurements:**

35 clinical features were used to cover all three phases of inpatient treatment (pre-, within-, post-treatment). Among these, sociodemographic characteristics, ICD-10 psychiatric diagnoses, previous detoxification treatments, and somatic measurements as well as inpatient treatment setting such as withdrawal medication, liver ultrasound, further information about the patients´ stay, and post-inpatient care were assessed. The four outcome dimensions included: continuous abstinence, abstinence at follow up, daily alcohol consumption, and days of abstinence after discharge.

**Findings:**

Six months after withdrawal treatment 46% of the patients achieved continuous abstinence. Socioeconomic, clinical and somatic features across the treatment timeline were analyzed and summarized into a multivariate signature associated with long-term treatment outcome. Thereby, the SPLS algorithm identified regular completion of withdrawal treatment, higher education, and employment status to be most strongly associated with a positive outcome. Alcohol-related hepatic and hematopoietic damage, number of previous withdrawal treatments and living in a shelter were most profoundly associated with a negative outcome.

**Conclusion:**

Conceiving treatment outcome as a multidimensional signature and moving beyond simple binary classifications of relapse versus abstinence may improve the understanding of relapse pathways and support more individualized treatment strategies.

## Introduction

Alcohol dependence exerts a profound and long-lasting negative impact on individual and socioeconomic levels (1, 2). One key challenge of alcohol dependence is not only reaching abstinence but managing it and minimizing the risk of relapse. Despite decades of research dedicated to understanding relapse risk factors, relapse rates often range over 50% within the first year after alcohol withdrawal treatment (3–6). In addition to major individual consequences, the high rate of relapse is a challenge for health care systems with limited resources and a need for stratified treatment allocation (7). Identified risk factors of relapse are, among others, younger age, lack of employment, drug use, previous therapy drop-outs, psychiatric comorbidities, addiction severity and craving (8–13).

Despite advances in the understanding of relapse pathways, the translational success has been limited. One underlying issue might be the methodical and conceptual heterogeneity of the studies investigating recovery from alcohol dependence and the lack of a clear and universally accepted definition of abstinence, with concepts ranging from absolute abstinence, point-prevalence abstinence to reduced drinking (14–16). Moreover, a binary conceptualization of abstinence might not reflect the complex, often multifaceted nature of an affected individual’s struggle with alcohol disease (17). Furthermore, study assessments mostly rely on clinical interviews and self-reports, while somatic features and therefore potential biomarkers have not been consistently established or validated (18). Finally, most studies employ univariate group-level comparisons or correlation analyses, which carry the advantage of clear and easily interpretable results, but are limited in their translational applicability to new cohorts or previously unseen individuals as well as in capturing more complex, distributed, and multi-faceted signatures (19).

The current body of literature shows that alcohol addiction and abstinence are complex phenomena with diverse contributing factors leading to possibly non-linear, multifaceted disease and relapse mechanisms. Therefore, a more precise clinical and neurobiological subtyping of patients suffering from alcohol dependence has been proposed to tackle the complexity of relapse risk assessment (20).

Recent studies have increasingly used machine learning approaches to predict adolescents’ alcohol use (21), the risk to develop alcohol dependence (22, 23), later drinking behavior (24) and alcohol withdrawal symptoms (25). Implementing machine learning approaches in this context could potentially entail a deep and multimodal understanding of the complex risk factors for disease progression as well as an individualized risk assessment, possibly allowing the development of tailored and more effective treatment strategies.

Hereby, we present a study that uses a diverse set of individual, socioeconomic, biological, and clinical pre-, within-, and post-treatment features to identify a multidimensional signature associated with relapse and analyzing long-term outcomes in alcohol dependence treatment.

**Figure 1:**
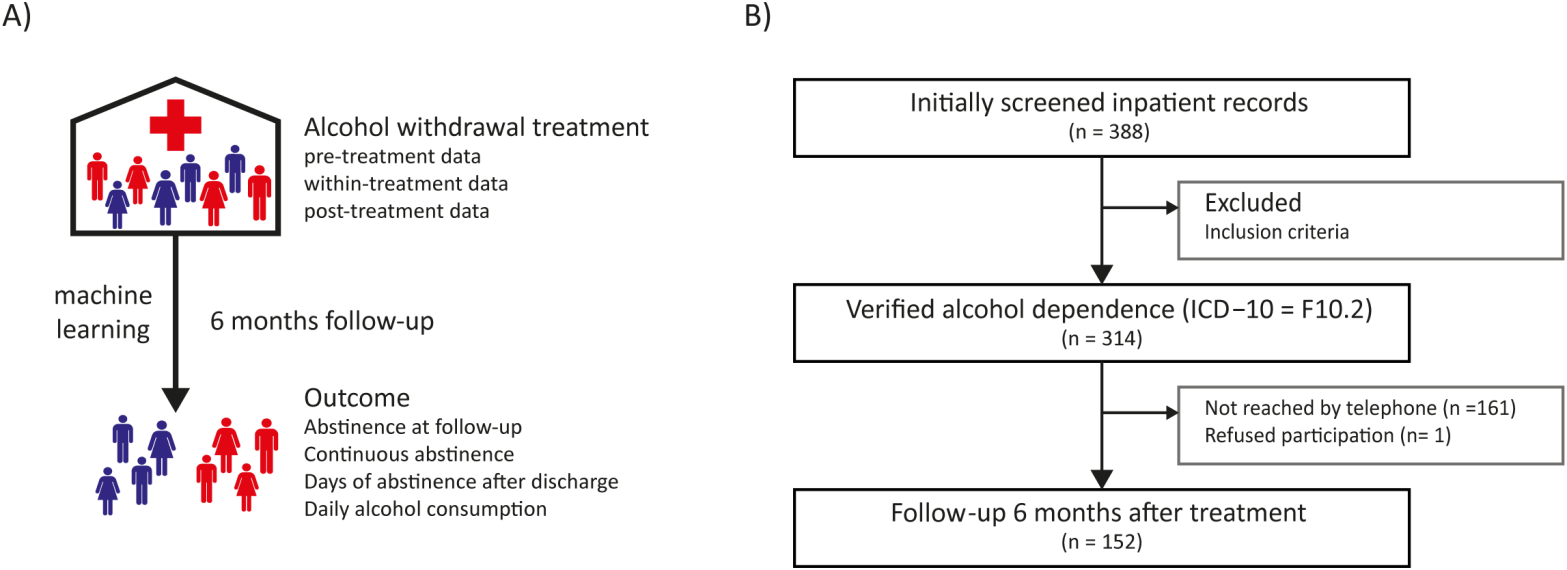
Schematic overview of the longitudinal study with 6-month follow-up and selection process. **(A)** Inpatients received alcohol withdrawal treatment. Biological, sociodemographic, and clinical data were categorized in data that are available before acute withdrawal treatment (pre-treatment data), data during the withdrawal treatment and post-treatment data. Patients were re-contacted 6 months after treatment to assess outcome parameters. Machine learning was applied to identify multivariate associations between pre-, within- and post-treatment data and 6-month outcome measures. **(B)** Initially 388 inpatient records were screened. 24 individuals did not meet the inclusion criteria. Of the remaining 314 individuals with a verified alcohol dependence (ICD-10: F10.2), 161 could not be reached for the follow-up phone call after 6 months, while one individual was reached, but refused participation in the phone call. Finally, 152 individuals were entered into the analysis.

In contrast to many previous studies, that focused on one single parameter to determine treatment outcome, we took an in-depth approach by assessing four different facets of alcohol-related outcome: continuous abstinence, abstinence at follow up, daily alcohol consumption, and days of abstinence after discharge (**Figure 1A**). Following a data-driven study design, we applied the multivariate Sparse Partial Least Squares (SPLS) algorithm to identify interpretable signatures using clinical data and outcome variables (26). We hypothesized that our approach would yield new insights into the complex and multifaceted nature of addiction that might serve as a template to adapt and enhance future clinical diagnostic and treatment algorithms.

## Methods

### Design and setting

This naturalistic longitudinal study with 6 months follow-up was approved by the local ethics committee of the Faculty of Medicine, LMU Munich, Germany (project number 585-16). The study design has been previously published by Raabe et al. (27). From March 18, 2016 to July 26, 2017 inpatients received standardized multimodal and multi-professional inpatient alcohol withdrawal treatment (28) at the Department of Psychiatry and Psychotherapy, University Hospital, LMU Munich, Munich, Germany. Inpatient alcohol withdrawal treatment is a multimodal and multi-professional inpatient detoxification and treatment program (28), which usually takes about 14–21 days. Inpatient alcohol withdrawal treatment is highly standardized and established in the inpatient addiction unit at the Department of Psychiatry and Psychotherapy, University Hospital, LMU Munich, Munich, Germany. Patients received medication as needed and acute withdrawal symptoms were treated in a standardized manner with benzodiazepines (oxazepam) and alpha-2 noradrenergic agonists (clonidine). Furthermore, patients received standardized doses of the anticonvulsant carbamazepine or levetiracetam upon a history of previous epileptic seizures. Abstinence during inpatient alcohol withdrawal treatment was monitored with breath analyzers for alcohol. Hence classical written informed consent includes a bias risk itself (29), we performed a modified version, that was approved by the local ethic committee. Two weeks before the 6 months follow-up assessment, former patients received written information to inform them about the study. Before the 6 months follow-up assessment was performed via phone by trained interviewers, successfully contacted patients were asked whether they agreed to participate in the study they were informed about 2 weeks before (obtained oral informed consent). In this study all patients received withdrawal treatment within a single clinical unit under the supervision of the same senior physician and treatment team.

### Assessment of clinical characteristics

A total of 35 features were evaluated as the predictor block (**Table 1**). These features were further categorized into 17 pre-, 12 within-, and 6 post-treatment features, containing both anamnestic and biological data. At admission, patients received a standard questionnaire based on the European Addiction Severity Index (EuropASI) (30). The pre-treatment features contained sociodemographic information (age, sex, education, employment, and family status, living situation) as well as information on previous withdrawal-related treatments and incidents (seizures, deliria) and comorbid psychiatric disorders or additional drug consumption. The within-withdrawal treatment features included breath alcohol at admission, liver disease detected by ultrasound, usage of withdrawal medication within first five days of treatment (clonidine, oxazepam) and further information regarding the inpatient stay (room occupancy type, total days in the hospital, type of discharge). Furthermore, we collected routine blood biomarkers within 72h after admission to assess alcohol-related hepatic and hematopoietic damage via the AST/ALT ratio (31) and the combined threshold criterion of AST/ALT > 1.0 and MCV > 90.0 fl. The combined threshold criterion has already been successfully employed to identify patients with alcohol dependence (32). Post-treatment features covered anti-relapse medication naltrexone and acamprosate (obtained from registry data), as well as participation in post-inpatient treatment such as admission to day clinics, self-help groups and long-term rehabilitation settings as well as retrospective assessment of treatment satisfaction using the Munich Patient Satisfaction Scale (33). All these data were assessed via self-report during the follow-up telephone interview.

**Table 1:** Clinical and demographic study sample.

Variable	All	Continuous abstinence	Abstinence at follow-up only	No abstinence at follow-up	H/χ^2^	P
N	152	70	49	33		
Pre-treatment						
Sex, % male	72.4	80.0	65.3	66.7	3.80^c^	0.91
Age, years	47.8 (12.8)	48.8 (13.2)	45.7 (13.1)	48.9 (11.6)	2.65^b^	0.92
Previous alcohol consumption, g/day	190.9 (105.6)	198.3 (116.6)	173.5 (84.2)	201.2 (109.7)	0.94^b^	0.96
Tobacco smoking, %	59.0	54.0	63.0	64.0	1.30^c^	0.95
Consumption of other drugs, %	30.0	26.0	39.0	24.0	2.94^c^	0.91
Comorbid psychiatric disorder, %	57.0	54.0	61.0	55.0	0.64^c^	0.97
Depressive disorder (ICD-10 F32/F33), %	41.4	41.4	40.8	42.4	2.89^c^	0.93
Anxiety disorder (ICD-10 F41), %	3.3	0.0	6.1	6.1	6.11^c^	0.63
Personality disorder (ICD-10 F60), %	8.6	5.7	12.2	9.1	4.27^c^	0.79
Level of education^a^	2.5 (1.2)	2.7 (1.2)	2.6 (1.2)	2.2 (1.1)	3.97^b^	0.90
Living in partnership/marriage, %	43.0	43.0	41.0	45.0	0.17^c^	0.99
Employment, %	47.0	53.0	45.0	36.0	2.55^c^	0.92
No. of previous withdrawal treatments	4.6 (6.6)	3.2 (3.6)	6.7 (9.0)	4.5 (6.5)	5.62^b^	0.59
Previous withdrawal delirium, %	8.0	7.0	6.0	12.0	1.08^c^	0.95
Previous withdrawal seizures, %	20.0	14.0	22.0	30.0	3.73^c^	0.91
LS: with family members/children, %	38.0	36.0	41.0	36.0	0.34^c^	0.98
LS: alone, %	51.0	53.0	49.0	48.0	0.25^c^	0.99
LS: shared flat, %	3.0	3.0	4.0	3.0	0.14^c^	0.99
LS: assisted living, %	3.0	3.0	4.0	0.0	1.31^c^	0.95
LS: shelter, %	6.0	6.0	2.0	12.0	3.61^c^	0.91
Within-treatment						
Breath alcohol at admission	0.74 (0.79)	0.65 (0.76)	0.72 (0.80)	0.95 (0.82)	3.73^b^	0.91
Liver damage, %	45.0	43.0	49.0	42.0	0.53^c^	0.97
AST/ALT ratio	1.21 (0.51)	1.17 (0.45)	1.18 (0.60)	1.34 (0.45)	5.83^b^	0.53
AST/ALT >1.0 & MCV >90.0 fl., %	45.0	40.0	39.0	67.0	7.71^b^	0.21
Cumulative oxazepam dosage [mg]	67.9 (94.1)	66.9 (82.6)	51.5 (82.8)	94.5 (125.1)	3.53^b^	0.91
Cumulative clonidine dosage [µg]	65.3 (159.6)	79.3 (174.6)	59.7 (175.2)	43.9 (86.4)	1.41^b^	0.94
Duration of withdrawal treatment (d)	16.6 (6.7)	17.3 (7.8)	15.2 (4.8)	17.2 (6.6)	3.05^b^	0.91
ROT: twin room, %	26.0	24.0	22.0	33.0	1.35^c^	0.95
ROT: five-bed room, %	48.0	44.0	55.0	45.0	1.46^c^	0.94
ROT: supervision room, %	13.0	13.0	12.0	12.0	0.02^c^	1.00
ROT: room change, %	14.0	19.0	10.0	9.0	2.49^c^	0.92
Regular treatment completion, %	82.0	89.0	73.0	79.0	4.59^c^	0.98
Post-treatment^1^						
Naltrexone treatment, %	15.0	11.0	14.0	24.0	2.91^c^	0.91
Acamprosate treatment, %	13.0	14.0	16.0	6.0	1.96^c^	0.93
Day clinic treatment, %	32.0	39.0	20.0	33.0	4.46^c^	0.90
Self-help group admission, %	29.0	30.0	37.0	15.0	4.54^c^	0.90
Long-term rehabilitation^2^, %	30.0	36.0	24.0	24.0	2.32^c^	0.92
Self-reported satisfaction	22.6 (3.9)	22.7 (3.8)	22.1 (4.2)	22.9 (3.7)	2.41^b^	0.92
Outcome						
Abstinent at follow-up, %	78.3	100.0	100.0	0.0	152.0^c^	<0.001
Continuous abstinence, %	46.1	100.0	0.0	0.0	152.0^c^	<0.001
Daily alcohol consumption (g/day)	20.0 (55.6)	0.0 (0.0)	0.0 (0.0)	92.2 (88.0)	148.0^b^	<0.001
Days of abstinence after discharge	113.9 (70.0)	177.3 (7.6)	55.8 (48.7)	65.6 (55.3)	116.0^b^	<0.001

Values are mean (SD) unless otherwise indicated. Continuous variables were compared across outcome groups using the Kruskal–Wallis H test, and categorical variables using the χ^2^ test. P values are false discovery rate (FDR)–corrected. Outcome groups are mutually exclusive: continuous abstinence; abstinence at follow-up only (abstinent at follow-up but not continuously abstinent); and no abstinence at follow-up. “Previous alcohol consumption” refers to the average daily alcohol intake reported by patients for the week prior to admission to withdrawal treatment. “Consumption of other drugs” was coded as a dichotomous variable indicating any non-opioid-substitution substance use prior to admission; frequency and specific substances were not recorded. LS, Living Situation; AST, Aspartate Transferase; ALT, Alanine Transaminase; MCV, Mean Corpuscular Volume; ROT, Room Occupancy Type. ^a^Education coded on ordinal scale (1–4). ^b^Kruskal–Wallis H test. ^c^χ^2^ test.

Since this was a naturalistic, single-center study, we included all variables and data that were readily available to us. We speculated that even variables such as the room occupancy type or other characteristics of the treatment facility might play a role in the long run. We did not conduct any feature selection, but rather acquired all data that was available to us and then employed a hypothesis-free data-driven approach to identify the most relevant pre-, within- and post-treatment features for long-term outcome. Therefore, we included well-established variables along with more speculative, understudied variables such as “room occupancy type”. During the treatment, patients were accommodated in either two-bed rooms, five-bed rooms, or a supervision room with camera (for severe cases), with the possibility of switching between room categories when availability allowed.

### Outcome assessment

The treatment outcome features were evaluated 6 months after discharge of alcohol withdrawal treatment by a follow up interview to assess both drinking habit and abstinence status. We used “continuous abstinence” and “abstinence at follow up” as binary parameters, and “daily alcohol consumption” as well as “days of abstinence after discharge” as continuous metric parameters. “Continuous abstinence” was defined as the patient’s self-reported maintenance of abstinence from the time of discharge from alcohol withdrawal treatment up to the follow-up interview. In our study, “abstinence at follow-up” was defined as uninterrupted abstinence during the 30 days preceding the follow-up interview (conducted six months after discharge from alcohol withdrawal treatment), using the Alcohol Timeline Followback (TLFB) method (34), where TLFB = 0 g of alcohol. For the metric parameter “daily alcohol consumption”, we calculated the alcohol intake in grams of alcohol during the Alcohol Timeline Follow Back assessment of 30 days. Alcohol consumption during follow-up was assessed using the Timeline Follow-Back (TLFB) method for the preceding 30 days, which was selected because this recall window is considered reliable for patient self-report; previous studies have demonstrated substantial agreement between self-reported alcohol use and biochemical measures while also acknowledging potential influences of recall bias and social desirability, highlighting the value of complementing self-reports with objective biomarkers in future studies (35–37). Finally, “days of abstinence after discharge” was assessed counting the days without any alcohol intake from the moment of being discharged from alcohol withdrawal treatment to the first relapse.

### Participants

The study took place between September 2016 and January 2018. Inclusion criteria were: 1) patients with a confirmed diagnosis of alcohol dependence (ICD-10: F10.2) according to the International Classification of Diseases and Related Health Problems 10th Revision (ICD-10), 2) treated in the inpatient addiction unit, and 3) age ≥ 18 years. Using electronic records, we identified 338 patients (71.3% male, 28.7% female, mean [SD] age: 46.7 [12.6] years) with a recorded diagnosis of alcohol dependence according to ICD-10. To exclude patients with a diagnosis other than alcohol dependence (e.g., multiple drug abuse [F19.2]), trained physicians re-assessed each patient’s diagnosis and excluded 24 patients with a diagnosis other than alcohol dependence (e.g., multiple drug abuse [F19.2]). Of the 314 eligible patients, 157 were successfully contacted at follow-up (50.0%). One person declined to participate in the follow-up interview and four other individuals had incomplete data required for the SPLS analysis and were therefore excluded, resulting in a final analytical sample of 152 participants. Thus, a total of 152 out of 314 (48.4%) former inpatients were contacted successfully 6 months [+/- 30 days] after inpatient alcohol withdrawal treatment and interviewed (**Figure 1B**). Baseline and within-treatment characteristics did not differ significantly between the study sample and patients lost to follow-up (**Supplementary Table 1**).

### Statistical analyses

Group-level differences were assessed using nonparametric tests (Kruskal-Wallis H test, χ^2^-Test). Autocorrelation analyses between the predictor and outcome dataset were computed using Spearman’s correlation coefficient (r). Analyses were false discovery rate–corrected for multiple testing at a significance threshold of q = 0.05 (38).

We used multimodal predictor and outcome data as input for the Sparse Partial Least Squares (SPLS) algorithm. Our predictor dataset contained 17 pre-treatment features, 12 within-treatment features as well as 6 post-treatment features, amounting to a total of 35 features. These features were selected due to their previously well-established association with alcohol addiction, treatment response and relapse (8, 39). The outcome dataset consisted of the four assessed alcohol related outcomes: continuous abstinence, abstinence at follow up, daily alcohol consumption, and days of abstinence after discharge. Given these two datasets, SPLS employs singular value decomposition to compute a latent variable (LV) capturing a specific associative effect between predictor and outcome data. For each dataset, the LV generates a vector with feature weights (values ranging from -1 to 1) measuring the covariance between the two datasets. Hence, the LV consists of paired multivariate profiles measuring how the predictor block features relate to the outcome features (Supplement). Another characteristic of SPLS is the enforcement of sparsity, whereby weights of zero are assigned to features that did not yield any relevant association. The weighting and selection of features is accomplished via l1- and l2-norm constraints, comparable to elastic net regularization (40), and controlled by a pair of hyperparameters.

**Figure 2:**
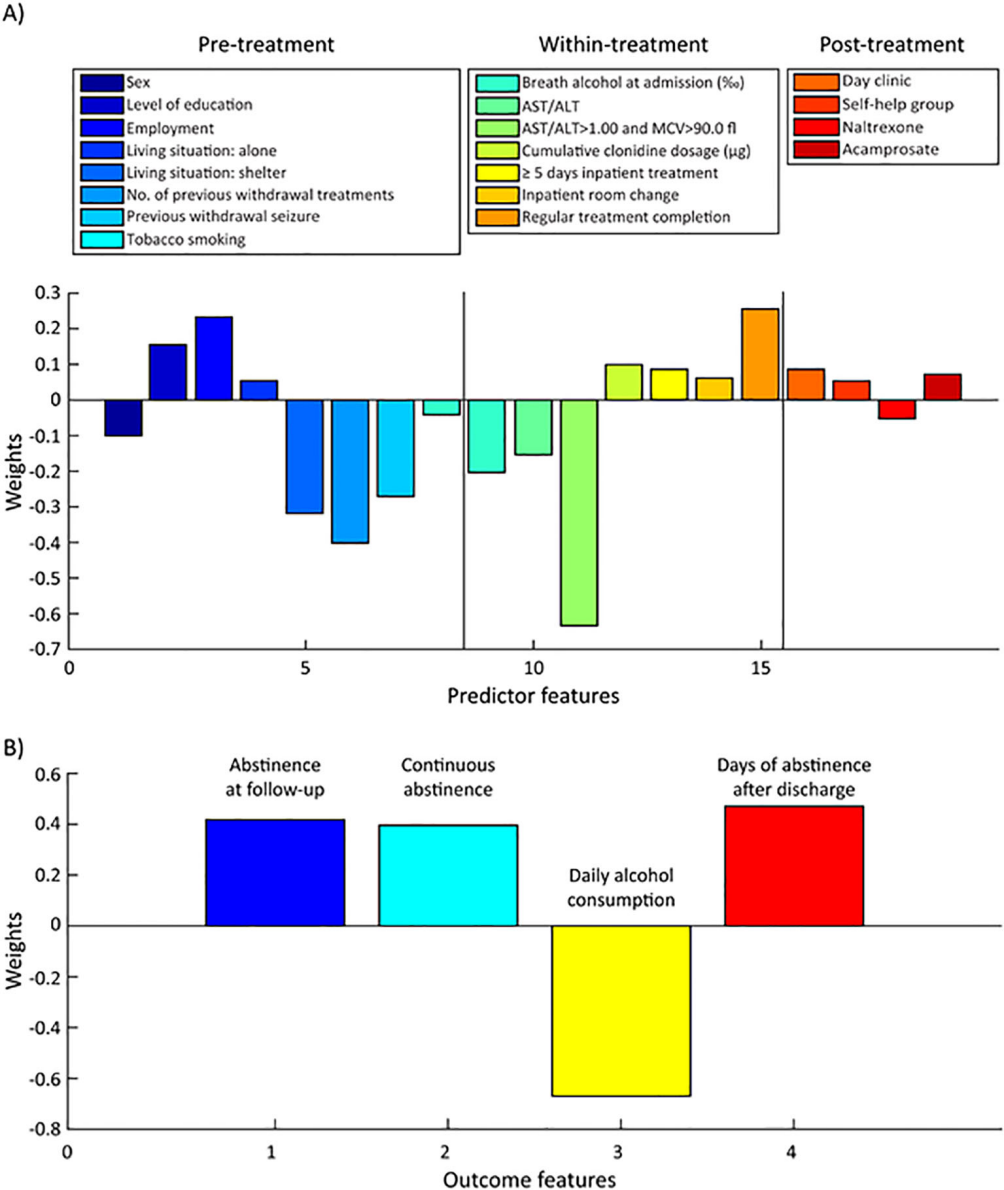
Multivariate signature between the predictor block and the outcome block. **(A)** Predictor features are separated into pre-, within- and post-treatment phase. **(B)** The favorable clinical outcome signature (abstinence-related) includes higher likelihood (positive weights) for abstinence at follow-up period, continuous abstinence, higher number of uninterrupted days of abstinence after withdrawal treatment, and lower average daily alcohol consumption, (negative weight).

We used the SPLS Toolbox developed by Popovic et al. (41), in which the SPLS algorithm is embedded in a nested cross-validation (NCV) framework, which robustly prevents information leakage between participants used for training and validating the models (42, 43) (**Supplementary Figure 1**). The SPLS Toolbox by Popovic et al. implements and extends the proposed SPLS framework by Monteiro et al. (26) by adding a nested cross-validation setup with embedded scaling and covariate correction, Procrustes rotation, bootstrapped feature-weight stability assessment, and hyperparameter optimization against non-sparse PLS. The SPLS Toolbox developed by Popovic et al. implements the SPLS framework of Monteiro et al. (26) with a nested cross-validation setup, embedded scaling/covariate correction, Procrustes rotation, bootstrap-based weight stability estimation, and hyperparameter optimization. The toolbox has been applied in several prior multimodal association studies (41, 44–47) and is available as open-source MATLAB code: https://github.com/dpopovic30/spls_toolbox_compiled. The SPLS algorithm employs singular value decomposition to derive latent variables (LVs), each comprising weight vectors that assign weights to every feature in the corresponding data matrix (**Supplementary Methods** - Sparse partial least squares algorithm). These weights encode the direction and magnitude of covariance between features. For each LV, participant-specific latent scores are obtained by projecting the LV weight vectors onto the data matrices, yielding individualized loadings, i.e., latent scores. The correlation between these latent scores—reflecting the covariance captured by the LV—served as the optimization criterion during hyperparameter optimization, i.e., model training. We implemented SPLS within a nested cross-validation framework (43) with group stratification and assessed significance via permutation testing. Feature stability was evaluated using bootstrap ratios (48), and significant LVs were iteratively removed via projection deflation (26). Multiple outcomes were analyzed jointly within a two-block sparse PLS (SPLS) framework: the predictor block contained pre-, within-, and post-treatment variables, and the outcome block comprised four abstinence measures (continuous abstinence, abstinence at follow-up, daily alcohol consumption, days of abstinence after discharge). SPLS estimates a multivariate association pattern between these two blocks, yielding latent variables with weights for both blocks; accordingly, only one model was trained and validated (**Figure 2**). Significance was assessed at the LV level via permutation testing, and no P value correction for multiple testing was required. We used 10 inner folds for hyperparameter optimization of the *l*_1_- and *l*_2_-norm constraints and 10 outer folds to test the optimized model against a previously held-out dataset. Before entering the SPLS analysis, all features were z-transformed within the NCV structure. The significance of each LV was assessed by comparing the performance of the optimized model against 5000 permutations of the dataset (26). If an LV proved significant, the respective covariance component was removed from the two datasets via projection deflation and the next LV was computed on the deflated datasets (49, 50). This process was repeated until an LV failed to reach significance (for further details, see **Supplementary Methods**).

## Results

### Group-level differences

On average, the clinical study population was 47.8 years (SD 12.8) old and 72.4% were male. The average daily alcohol consumption before treatment was 190.9 g/day, patients had experienced 4.6 (SD 6.6) previous withdrawal treatments and received on average 16.6 days (SD 6.7 days) of inpatient treatment in this study (**Table 1**). At the 6-month follow-up, 119 patients (78.3%) were abstinent at follow-up. Of these, 70 patients (46.1% of the total sample) had maintained continuous abstinence from discharge until follow-up, while 49 patients (32.2%) were abstinent at follow-up but had relapsed at least once during the follow-up period. The remaining 33 patients (21.7%) were not abstinent at follow-up. On average, patients remained abstinent for 113.9 days (SD 70.0) after discharge from withdrawal treatment. Across the entire sample, the average daily alcohol intake at follow-up was 20.0 g/day (SD 55.6 g/day). Using group-level statistical analyses, we did not observe any significant differences between the outcome groups (continuous abstinence, abstinence at follow-up only, and no abstinence at follow-up) with respect to pre-, within-, or post-treatment variables (**Table 1**; **Supplementary Figure 2**).

Post-treatment interventions were heterogeneous and not mutually exclusive. A supplementary analysis showed that 54 patients (35.5%) did not receive any structured post-treatment intervention, while 58 (38.2%) received one, 30 (19.7%) received two, and 10 (6.6%) received three or more interventions (**Supplementary Table 2**). An exploratory analysis examined the relationship between the number of post-treatment interventions and outcome category. Patients were grouped into those receiving no intervention, one intervention, or two or more interventions. While patients without structured aftercare were somewhat more frequent in the non-abstinent group, the association between the number of post-treatment interventions and outcome did not reach statistical significance (χ^2^ = 4.21, p = 0.38; **Supplementary Table 3**). Likewise, exploratory univariate analyses examining the association between individual post-treatment interventions and outcome category showed no statistically significant relationships after correction for multiple testing (**Supplementary Table 4**).

### SPLS results

SPLS analysis of all 152 individuals yielded one significant LV (LV1, *P* = 0.0305, r = 0.798, R^2^ = 0.637), representing a specific multivariate outcome signature (**Figure 2**) that contains abstinence at follow-up, continuous abstinence, achieved days of abstinence after discharge (all weighted positive), and lower daily alcohol intake (weighted negative), meeting the criteria of a favorable clinical outcome.

Regarding the pre-treatment features, the model links higher education level (feature weight: 0.155), positive employment status (0.233), and living alone (0.054) with a positive treatment outcome pattern. The positive outcome pattern was reflected in positive weights, i.e., higher rates of abstinence at follow-up and continuous abstinence, higher number of abstinent days after discharge, and lower average daily alcohol consumption (**Figure 2B**). On the other hand, female sex (-0.100), living in a shelter (-0.319), higher number of previous alcohol withdrawal treatments (-0.402), previous withdrawal seizures (-0.271) and tobacco consumption (-0.041) were associated with a negative treatment outcome pattern (lower rate of abstinence at follow-up and continuous abstinence, lower number of abstinent days after discharge, higher average daily alcohol consumption). With respect to within-treatment features, higher cumulative clonidine dosage (0.099), inpatient treatment duration over 5 days (0.086), inpatient room change (0.062), as well as regular completion of the inpatient alcohol withdrawal treatment program (0.255), were associated with a positive treatment outcome pattern. Higher breath alcohol at admission (-0.203), higher AST/ALT ratio (-0.154), and fulfilling the blood-based threshold combination criterion of AST/ALT > 1.0 and MCV > 90.0 fl. for alcohol-related hepatic and hematopoietic damage (-0.634) were associated with a negative treatment outcome pattern. Among the post-treatment features, participating in day clinic treatment (0.085) or self-help group sessions (0.053) as well as usage of acamprosate (0.072) were related to positive treatment outcome pattern, while intake of naltrexone (-0.052) was related to a negative treatment outcome pattern.

**Figure 3:**
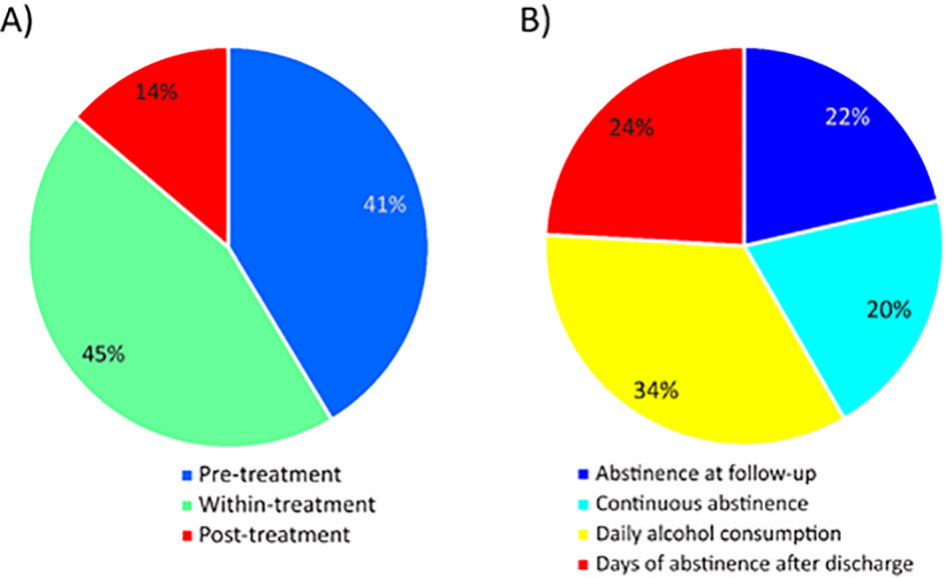
Distribution of predictor block and outcome block feature weights. **(A)** The pie chart illustrates the proportion (accumulated weights) of the significant pre-, within- and post-treatment features within the SPLS model. **(B)** The pie chart illustrates the proportion (weights) of the abstinence and relapse features within the SPLS model.

To estimate the relative contribution of both data blocks in the overall signature, we computed the mean of absolute feature weights (**Figure 3**). Hence, the signature consisted of 45% pre-, 41% % within- and 14% post-treatment feature weights. The outcome pattern featured relative weights of 34% of daily alcohol consumption, 24% days of abstinence after discharge as well as 22% and 20% for abstinence at follow-up and continuous abstinence after discharge, respectively.

## Discussion

We aimed to identify a multidimensional signature of long-term outcome after inpatient alcohol withdrawal treatment. By incorporating multimodal data from pre-, within-, and post-treatment phases and conducting a multivariate machine learning approach, we were able to generate a comprehensive signature linking sociobiographic, clinical and somatic features to a multi-faceted pattern of abstinence-related outcomes six months after inpatient withdrawal treatment. The SPLS approach captured approximately 63% of the shared covariance between the predictor and outcome blocks, indicating that the identified multivariate signature explains the majority of the shared variance between clinical and long-term outcome measures.

Multiple measures of long-term treatment outcome after inpatient withdrawal treatment were used since alcohol-related outcomes lack a universal definition. While abstinence is usually defined as complete avoidance of any alcohol consumption, other conceptualizations use “sustained or continuous abstinence” (meaning not drinking at all over a certain period) (51, 52), “reduced drinking” (drinking smaller amounts of alcohol than recorded before the alcohol withdrawal treatment) (16, 53), and “point-prevalence abstinence” (no alcohol use in e.g., the past 30 days at a certain follow-up) (54–56).

Our aim was to cover different types of outcomes since nature of relapse and abstinence is heterogeneous. We interpreted the pattern of continuous abstinence after discharge from inpatient withdrawal treatment, abstinence at follow up, lower daily alcohol consumption and higher number of continuous abstinent days after discharge as positive outcome and lack of continuous abstinence after discharge from alcohol withdrawal treatment, no abstinence at follow up, higher daily alcohol consumption and lower number of continuous abstinent days after discharge as negative outcome.

Pre-treatment features associated with positive outcomes included male sex, employment, higher educational attainment, and living alone. In contrast, features associated with poorer outcomes included living in a shelter, a higher number of previous withdrawal treatments, a history of withdrawal seizures, and tobacco use. These findings are consistent with prior studies associating employment with better outcomes in terms of abstinence and alcohol consumption (9, 12, 57, 58).

The association of smoking with poorer outcomes was also supported by previous research, which found tobacco use to be linked with greater severity of alcohol dependence (59) and earlier risk of alcohol relapse (60). While a higher number of previous withdrawal treatments, being reflective of more severe cases and higher degrees of chronification, has already been described as a negative risk factor for future abstinence (61), our results also suggest that previous withdrawal seizures are linked to negative treatment outcomes.

Among within-treatment features, higher cumulative clonidine dosage, an inpatient stay longer than 5 days, inpatient room change and completion of the withdrawal treatment (i.e., regular discharge) were associated with a favorable outcome. Higher breath alcohol at admission as well as alcohol-related hepatic and hematopoietic damage based on blood-based biomarkers (AST/ALT ratio, combined threshold criterion of AST/ALT > 1 and MCV > 90fl.) were related to poorer outcomes.

We identified the blood biomarker-derived threshold criterion for alcohol-related hepatic and hematopoietic damage (AST/ALT > 1.0 and MCV > 90.0 fl.) as the feature most strongly associated with a negative outcome. Until now this criterion was used for the identifications of patients with alcohol dependence among general somatic patient populations (32), and our findings suggest that it could potentially serve as a possible biomarker to identify the most vulnerable patients with alcohol dependence (27). Positive therapy motivation has been found to be associated with a better treatment outcome (62, 63), which is aligned with our findings of participation in day clinic and self-help groups and the resulted positive outcome pattern. Lower breath alcohol at admission, which correlates with daily alcohol consumption and severity of the addiction (64), longer duration of inpatient treatment (>5 days), as well as regular completion of the withdrawal treatment (65) were linked to beneficial treatment outcomes. Additionally, our results indicated that higher cumulative clonidine dosage was associated with a beneficial outcome. Thus, the increased clonidine dosage could be a proxy for more proactive treatment of early withdrawal symptoms. The inpatient room change as a positive factor in our finding could similarly reflect the intent to adapt the inpatient setting to the individual’s needs with respect to better sleeping environment, a better chemistry between the inpatients or other interpersonal factors. These findings would support a more proactive and individualized approach to withdrawal treatment, specifically in the early stages of admission.

In the post-treatment phase, participation in day clinic programs and self-help groups showed positive weights within the multivariate SPLS pattern and were therefore associated with the favorable outcome signature. This matches previous work, underlining the benefits of regular attendance at post-acute treatment to support abstinence (66, 67). Also related to positive outcomes was the use of the anti-craving medication acamprosate, while naltrexone use was linked to poorer outcomes. The opposite associations observed for acamprosate and naltrexone should be interpreted with caution, as medication allocation was not randomized and may reflect confounding by indication. While acamprosate is typically prescribed to patients who have already achieved abstinence, naltrexone is often used in individuals with ongoing drinking or higher relapse risk. Therefore, this pattern could reflect individual prescribing patterns rather than causal treatment effects (68–70). However, prescribing variability was likely limited as all patients were treated within a single clinical unit under the supervision of the same physician and treatment team.

Still, post-treatment features should be interpreted with caution, as treatment allocation was not randomized and may reflect underlying relapse risk or clinical decision-making rather than causal treatment effects. In addition, outcomes were assessed at a single six-month follow-up time point, limiting the ability to determine the temporal relationship between post-discharge interventions and drinking outcomes. Additional exploratory analyses examining both the number and type of post-treatment interventions showed no significant association with outcome category (**Supplementary Tables 3–S4**), suggesting that these variables alone do not account for the multivariate associations identified by the SPLS model.

Overall, the pattern linked to long-term treatment response was diverse and included socioeconomical, clinical, and biological facets, while the outcome pattern covered not only binary, but also continuous dimensions of abstinence and drinking behavior. Thus, investigations into alcohol dependence and treatment might benefit from evolving from binary outcome parameters, such as abstinence or no abstinence, to outcome patterns based on multiple parameters. This might more accurately reflect the affected individual’s challenging circumstances and could therefore enable more precise and tailored interventions.

The integration of machine learning approaches, such as sparse partial least squares (SPLS), enables the detection of complex associations between predictor and treatment blocks. These methods can contribute to personalized, tailored treatments (71) and interventions (72) in alcohol dependence. The treatment outcome signature was composed of 45% pre-treatment, 41% within-treatment, and 14% *post-treatment* feature weights (**Figure 3A**). This distribution underscores the importance of initiating inpatient alcohol withdrawal treatment as early as possible to prevent or mitigate further psychosocial and somatic deterioration. Once treatment has commenced, particular emphasis must be placed on optimizing the course and ensuring successful completion of inpatient alcohol withdrawal treatment, as this phase represents a critical window for therapeutic intervention. As most features were identified during pre- and within-treatment phases, this may reflect greater measurement precision at these times, where many features were assessed and validated by the attending clinician. However, post-treatment factors were potentially subject to unmeasured influences and solely assessed via self-report through telephone calls.

Several identified outcome-related features have direct clinical relevance. The combined biomarker criterion of an AST/ALT ratio > 1.0 and MCV > 90.0 fL represents a readily available and cost-effective indicator that could complement established screening tools such as AUDIT or AUDIT-C in both inpatient and outpatient settings. Particularly when self-report data are limited by recall or social desirability bias, integrating such laboratory markers into digital decision-support tools may improve early detection of high-risk individuals and facilitate timely medical and psychosocial interventions. In addition, contextual inpatient withdrawal treatment factors such as room occupancy and the possibility of room changes may influence subjective well-being and treatment adherence. These potentially modifiable environmental aspects highlight that relapse risk is not solely patient-inherent but may also be shaped by individual, patient-centric characteristics of care. At an interpersonal and treatment-environment level, such modifiable structural factors could contribute to improved treatment adherence and completion rates. Assessing and addressing these features might help clinicians to identify actionable targets within the treatment setting itself.

The study is mainly limited by being conducted at a single center with a limited sample size and follow-ups. Increasing both the sample size and the number of centers, as well as conducting more long-term follow-ups would certainly increase the validity and generalizability of the findings. Yet, 6 months outcomes have proven to be good predictors for 5-year outcomes (73). In addition, complementing our outcome-related features with in-depth neurobiological assessment such as MRI or EEG may help in gaining deeper understanding of pathogenesis of alcohol dependence, relapse mechanisms and its treatment. Furthermore, the outcome features were derived from self-reports and telephone interviews, which carries the risk of subjective distortions (74). The reported daily alcohol consumption could be biased by the individual capacity and will to recall. Still, previous work could confirm the reliability of self-reports, which closely correlate with blood biomarkers (75–77). These issues should be addressed by larger-scale studies, employing a multi-center, multi-modal approach to further validate and expand our current findings. Moreover, in current international diagnostic frameworks, including the DSM-5 and the World Health Organization’s classifications, the term Alcohol Use Disorder (AUD) encompasses the full spectrum of alcohol-related pathology. This also includes syndromes, which were previously referred to as alcohol dependence, as well as many less severe forms of pathological alcohol consumption. However, only patients with a diagnosis of full-blown alcohol dependence (ICD-10: F10.2) were included in the study. Hence, our findings cannot be generalized for all AUD patients since our study focused mainly on a group of severely ill, highly addicted patients in need and willing to undergo alcohol withdrawal treatment. Future studies should aim to develop models that take into account the full spectrum of AUD to increase scalability and generalizability. Moreover, a more in-depth gradual assessment of pathological alcohol consumption, such as AUDIT scores, would offer further insights and should be included in future studies on this topic. Furthermore, future studies would benefit from additional in-person follow-up assessment, that would allow for the collection of biomarkers such as carbohydrate-deficient transferrin (CDT), ethyl glucuronide (EtG) or phosphatidylethanol (PEth) to enhance the objective quantification of alcohol intake over the preceding month. Although the European Addiction Severity Index (EuropASI) questionnaire was completed at admission as part of routine clinical intake, composite scores were not calculated and the instrument was not re-administered during the brief telephone follow-up interviews, and therefore addiction severity measures could not be included in the analyses. Additionally, the follow-up response rate was moderate, with a final analytic sample comprising 152 of 314 eligible patients (48.4%); although baseline and within-treatment characteristics did not differ significantly between participants and those lost to follow-up, the possibility of residual non-response bias due to unmeasured factors cannot be fully excluded. Finally, since SPLS models covariance structure rather than optimizing individual-level predictive accuracy, the present findings should be interpreted as an interpretable multivariate association signature rather than a validated risk prediction tool.

To our knowledge, this is the first study to examine relapse risk factors and long-term outcome of inpatient alcohol withdrawal treatment using a data driven, unsupervised machine learning approach and a multi-faceted approach to withdrawal treatment outcomes. We found a comprehensive clinically translatable signature, which linked individual and personal factors, as well as biological measurements and environmental features to long-term outcome after alcohol withdrawal treatment. Modifiable features—such as employment status, living situation, and the duration and completion of withdrawal treatment—proved to be most strongly linked to future relapse and abstinence, and could therefore be prioritized in the clinical setting. Furthermore, anamnestic information regarding previous withdrawal treatments and withdrawal related seizures, as well as breath alcohol at admission, blood biomarkers (liver enzymes, blood count alterations), and early need for withdrawal related medication could potentially lead to a better and more detailed assessment of at-risk patients who might demand increased medical attention during withdrawal treatment.

## Conclusion

Our findings underscore that long-term outcomes following alcohol withdrawal treatment are shaped by a multidimensional interplay of sociodemographic, clinical, and biological factors spanning all stages of care. By applying a machine learning framework, we identified a clinically interpretable signature that extends beyond binary relapse definitions and captures the complexity of recovery trajectories in alcohol dependence. This multidimensional perspective points to modifiable factors that can be addressed within routine psychiatric care. Embedding such tools into clinical pathways could help allocate resources more efficiently, guide personalized intervention strategies, and ultimately improve long-term recovery rates. Future multi-center studies with extended follow-up and integration of neurobiological measures are warranted to validate these findings and to advance precision medicine approaches in addiction psychiatry.

## Data Availability

The original contributions presented in the study are included in the article/**Supplementary Material**. Further inquiries can be directed to the corresponding author.
