# Supplementary material for: The patterns of relapse and abstinence: using machine learning to identify a multidimensional signature of long-term outcome after inpatient alcohol withdrawal treatment
Source: Front Psychiatry. 2026 Apr 17;17:1683069. doi: 10.3389/fpsyt.2026.1683069 (PMC13132820; doi:10.3389/fpsyt.2026.1683069)
Supplement: Supplementary file 1 [file DataSheet1.docx]

Supplemental information

The patterns of relapse and abstinence: Using machine learning to identify a multidimensional signature of long-term outcome after inpatient alcohol withdrawal treatment

Raabe, Brechtel et al.

**Content**

[Supplementary Tables 2](#_Toc223869768)

[Supplementary Figures 4](#_Toc223869769)

[Supplementary Methods 6](#_Toc223869770)

[Sparse partial least squares algorithm 6](#_Toc223869771)

[Machine learning framework 7](#_Toc223869772)

[References 10](#_Toc223869773)

# Supplementary Tables

**Table S1: Clinical and Demographic Differences between the Study Sample and the Sample lost to follow-up**.

Values are mean (SD) unless otherwise indicated. Continuous variables were compared between the study sample and the sample lost to follow-up using the Kruskal–Wallis H test, and categorical variables using the χ² test. P values are false discovery rate (FDR)–corrected. “Previous alcohol consumption” refers to the average daily alcohol intake reported by patients for the week prior to admission to withdrawal treatment. “Consumption of other drugs” was coded as a dichotomous variable indicating any non-opioid-substitution substance use prior to admission; frequency and specific substances were not recorded.

Abbreviations: LS, Living Situation AST, Aspartate Transferase ALT, Alanine Transaminase MCV, Mean Corpuscular Volume ROT, Room Occupancy Type. ᵃ Education coded on ordinal scale (1–4). ᵇ Kruskal–Wallis H test. ᶜ χ² test.

| **Variable** | **Study sample (n = 152)** | **Lost to follow-up (n = 162)** | **H/χ²** | **P** |
| --- | --- | --- | --- | --- |
| N | 152 | 162 | — | — |
| Pre-Treatment |  |  |  |  |
| Sex, % male | 72 | 70 | 0.14ᶜ | 0.92 |
| Age, years | 47.82 (12.84) | 46.31 (12.59) | 1.02ᵇ | 0.82 |
| Previous alcohol consumption, g/d | 190.96 (105.64) | 205.17 (132.48) | 1.18ᵇ | 0.82 |
| Tobacco smoking, % | 59 | 61 | 0.11ᶜ | 0.92 |
| Consumption of other drugs, % | 30 | 33 | 0.23ᶜ | 0.92 |
| Comorbid psychiatric disorder, % | 57 | 55 | 0.09ᶜ | 0.92 |
| Level of educationᵃ | 2.53 (1.20) | 2.49 (1.17) | 0.06ᵇ | 0.92 |
| Living in partnership/marriage, % | 43 | 41 | 0.08ᶜ | 0.92 |
| Employment, % | 47 | 45 | 0.07ᶜ | 0.92 |
| No. of previous withdrawal treatments | 4.59 (6.57) | 4.83 (6.94) | 0.08ᵇ | 0.92 |
| Previous withdrawal-related delirium, % | 8 | 9 | 0.05ᶜ | 0.92 |
| Previous withdrawal-related seizures, % | 20 | 21 | 0.02ᶜ | 0.96 |
| LS: with family members/children, % | 38 | 40 | 0.09ᶜ | 0.92 |
| LS: alone, % | 51 | 49 | 0.07ᶜ | 0.92 |
| LS: shared flat, % | 3 | 3 | 0.00ᶜ | 0.96 |
| LS: assisted living, % | 3 | 2 | 0.20ᶜ | 0.96 |
| LS: shelter, % | 6 | 6 | 0.00ᶜ | 0.96 |
| Within-treatment |  |  |  |  |
| Breath alcohol at admission | 0.74 (0.79) | 0.82 (0.86) | 0.71ᵇ | 0.82 |
| Liver damage, % | 45 | 47 | 0.11ᶜ | 0.92 |
| AST/ALT ratio | 1.21 (0.51) | 1.24 (0.55) | 0.15ᵇ | 0.92 |
| AST/ALT > 1.00 & MCV > 90.0 fl., % | 45 | 47 | 0.12ᶜ | 0.92 |
| Cumulative oxazepam dosage [mg] | 67.89 (94.09) | 70.52 (98.13) | 0.07ᵇ | 0.92 |
| Cumulative clonidine dosage [µg] | 65.30 (159.57) | 61.12 (152.44) | 0.04ᵇ | 0.92 |
| Duration of withdrawal treatment (d) | 16.63 (6.72) | 17.01 (7.21) | 0.12ᵇ | 0.92 |
| ROT: twin room, % | 26 | 25 | 0.04ᶜ | 0.92 |
| ROT: five-bed room, % | 48 | 50 | 0.07ᶜ | 0.92 |
| ROT: supervision room, % | 13 | 12 | 0.03ᶜ | 0.96 |
| ROT: room change, % | 14 | 13 | 0.03ᶜ | 0.96 |
| Regular treatment completion, % | 82 | 79 | 0.39ᶜ | 0.92 |

**Table S2: Distribution of post-treatment interventions.**

Depicted are the numbers of post-treatment interventions per individual, grouped as 0, 1, 2 and ≥3.

| **Number of post-treatment interventions** | **Participants (N)** | **%** |
| --- | --- | --- |
| 0 | 54 | 35.5 |
| 1 | 58 | 38.2 |
| 2 | 30 | 19.7 |
| ≥3 | 10 | 6.6 |
| **Total** | **152** | **100.0** |

**Table S3: Outcome by number of post-treatment interventions.**

No statistically significant association between number of post-treatment interventions and outcome category was found (χ² = 4.21, p = 0.38 (FDR corrected)).

| **Number of interventions** | **Continuous abstinence** | **Abstinence at follow-up only** | **No abstinence at follow-up** | **Total** |
| --- | --- | --- | --- | --- |
| **0** | 20 (28.6%) | 18 (36.7%) | 16 (48.5%) | 54 |
| **1** | 29 (41.4%) | 19 (38.8%) | 10 (30.3%) | 58 |
| **≥2** | 21 (30.0%) | 12 (24.5%) | 7 (21.2%) | 40 |
| **Total** | 70 | 49 | 33 | 152 |

**Table S4: Association between post-treatment interventions and outcome category.**

Depicted are the group-level tests between different types of post-treatment intervention and all three types of outcome in the study sample.

| **Post-treatment intervention** | **Continuous abstinence** | **Abstinence at follow-up only** | **No abstinence at follow-up** | **χ²** | **P** |
| --- | --- | --- | --- | --- | --- |
| **N** | **70** | **49** | **33** |  |  |
| Naltrexone | 8 (11.4%) | 7 (14.3%) | 8 (24.2%) | 2.91 | 0.91 |
| Acamprosate | 10 (14.3%) | 8 (16.3%) | 2 (6.1%) | 1.96 | 0.93 |
| Day clinic treatment | 27 (38.6%) | 10 (20.4%) | 11 (33.3%) | 4.46 | 0.90 |
| Self-help group participation | 21 (30.0%) | 18 (36.7%) | 5 (15.2%) | 4.54 | 0.90 |
| Long-term rehabilitation | 25 (35.7%) | 12 (24.5%) | 8 (24.2%) | 2.32 | 0.92 |

Supplementary Figures


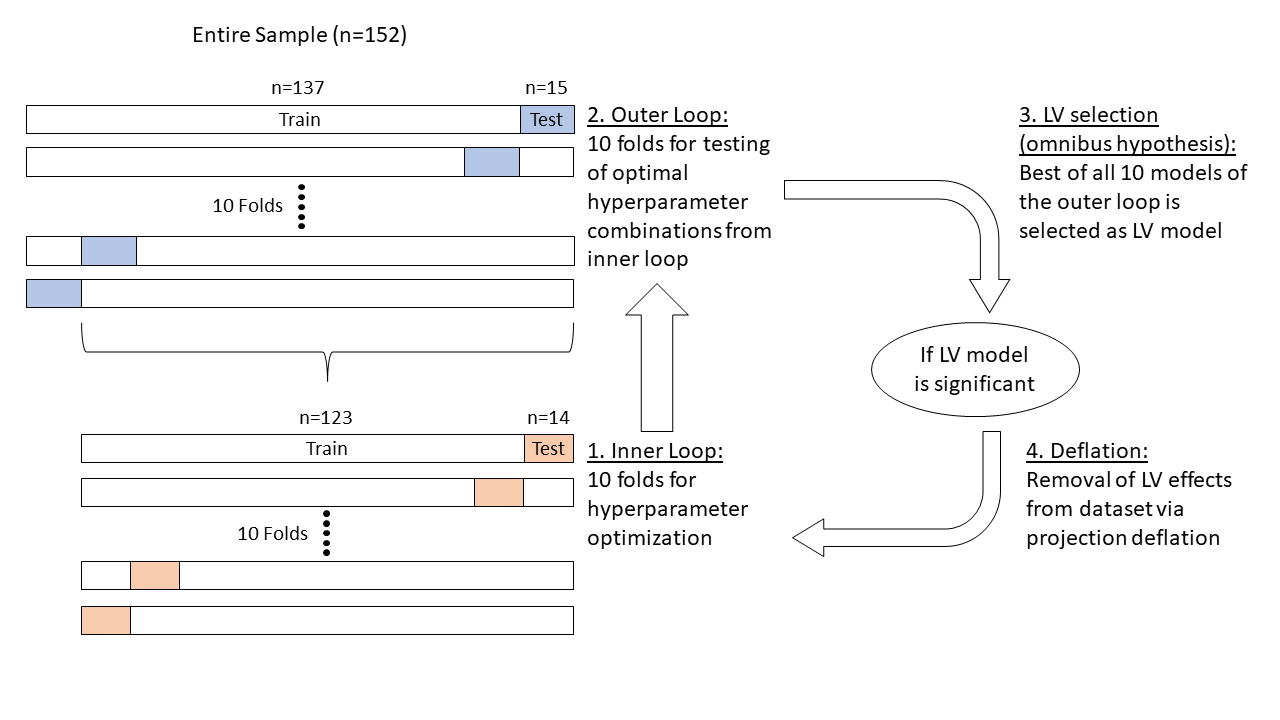


**Supplementary Figure S1: Nested cross-validation and external validation framework.**

Depicted is the nested cross-validation (NCV) framework with 10x10 folds on the CV2 and CV1 level. Hyperparameter optimization of c_u_ and c_v_ is performed on the CV1 level, whereas testing of the optimized model is done on the CV2 level. The best model of all 10 CV2 iterations is chosen as the LV model (in accordance with the omnibus hypothesis). If an LV proves significant against 5000 permutations, its effects are removed from the dataset via projection deflation, after which the next LV is computed. Adapted from Popovic et al. (1) and reprinted with permission.


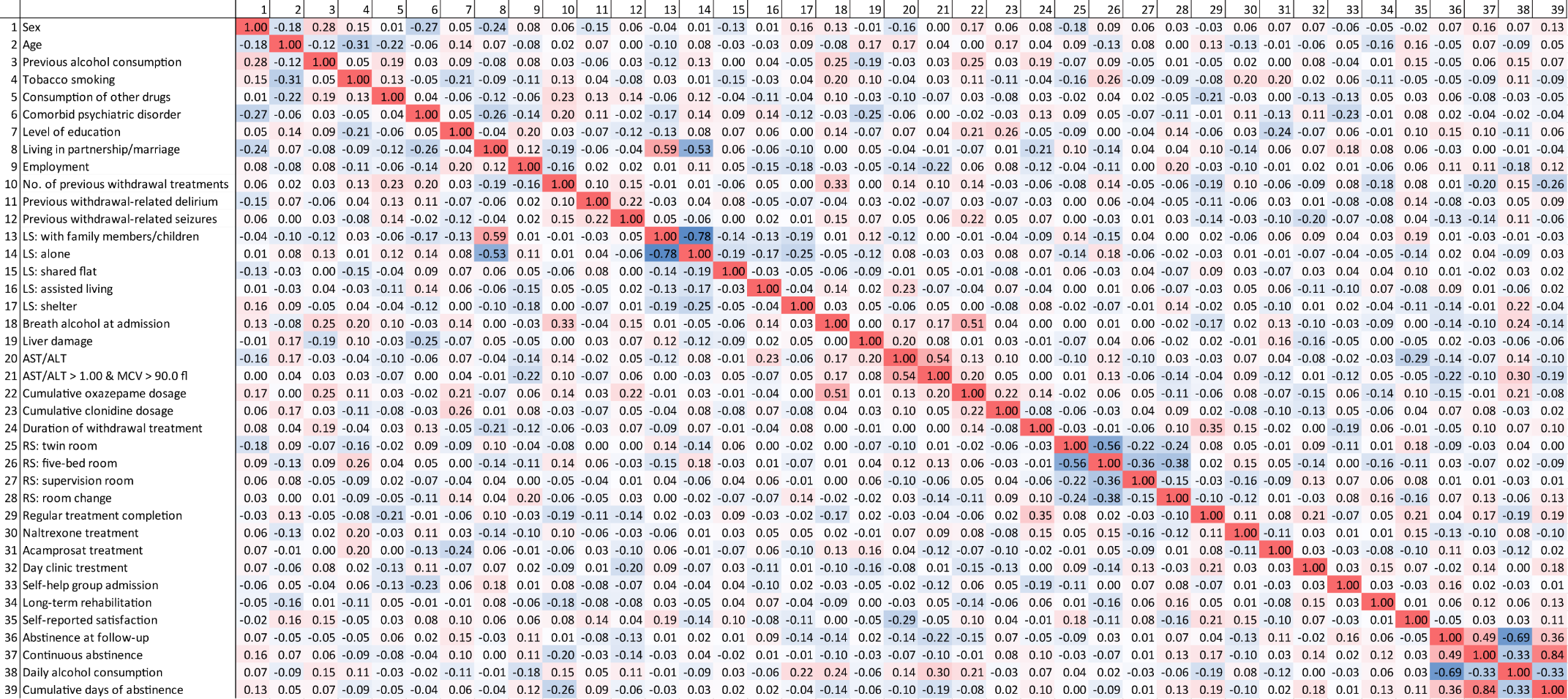


**Supplementary Figure S2: Autocorrelation Spearman’s coefficients of predictor and outcome block features.**

Depicted are Spearman correlation coefficients (r), with warmer colors indicating positive and cooler color scheme indicating negative coefficients. Abbreviations: LS, living situation; RS, room setting.

# Supplementary Methods

## Sparse partial least squares algorithm

The Sparse Partial Least Squares (SPLS) algorithm used in this analysis follows the original publication of Monteiro et al. (2) and has been described in a previous publication of the authors (1). Partial Least Squares (PLS) requires two data matrices $X$ and $Y$ as inputs. In our study, $X$ contains predictor information, while $Y$ contains outcome information. $n$ is the number of study participants; $p$ is the number of predictor features and $q$ is the number of outcome features. PLS provides insights into the relationships between different measures (i.e., views) from the same individuals. PLS identifies a latent space containing the relevant information in both views by finding pairs of weight vectors (generally called $u$ and $v$) which maximize the covariance between the projections of the two views (3):

1. ${maximize}_{{\|u\|}_{2}={\|v\|}_{2}=1} Cov\left( Xu,Yv \right)= {maximize}_{{\|u\|}_{2}={\|v\|}_{2}=1} u^{T}X^{T}Yv$

The weight vector pair is also referred to as a latent variable (LV) as it explains one specific associative effect between the two different views. Specifically, the weight vectors assign weights to each feature in the predictor and the outcome dataset, thus visualizing which features are associated with each other as well as the direction and the strength of this multivariate association. Hence, by studying this latent space, one can learn about the underlying relationship between predictor and outcome blocks (2).

In contrast to regular PLS, SPLS additionally enforces sparsity on the weight vectors $u$ and $v$ through hyperparameters $c_{u}$ and $c_{v}$. $c_{u}$ and $c_{v}$ are control the $l_{1}$-norm constraints of $u$ and $v$, respectively. The $l_{1}$-norm constraints impose sparsity, which means that the lower the values of $c_{u}$ and $c_{v}$ are, the higher the sparsity in the respective view is (4). This leads to the following optimization problem:

1. ${maximize}_{u,v} u^{T}X^{T}Yv subject to {\|u\|}_{2}^{2}\leq1, {\|v\|}_{2}^{2}\leq1, {\|u\|}_{1}\leq c_{u}, {\|v\|}_{1}\leq c_{v}$

Yet, this approach alone would only select up to$n$ features if $p>n$. Additionally, it would remove features which might be relevant for the model but are correlated with other features which are already included. Thus, Zou and Hastie addressed this issue by adding the $l_{2}$-norm constraints (5). For both $l_{1}$-norm and $l_{2}$-norm constraints to be active, the values of the hyperparameters must be between 1 and the square root of the number of features in the respective matrices. Therefore, the hyperparameter space is updated:

1. $1\leq c_{u} \leq\sqrt{p} , 1\leq c_{v}\leq\sqrt{q}$

Using the hyperparameter space of equation 3) and solving the optimization problem of equation 2) leads to the following SPLS algorithm steps as described in Monteiro et al. (2):

1. Let $C\leftarrow X^{T}Y$
2. Initialize $v$ to have ${\|v\|}_{2}=1$
3. Repeat until convergence:
   1. Update $u$:
      1. $u\leftarrow Cv$
      2. $u\leftarrow\frac{S\left( u, \Delta_{u} \right)}{{\|S\left( u, \Delta_{u} \right)\|}_{2}}$ , where $\Delta_{u}=0$ if this results in ${\|u\|}_{1}\leq c_{u}$, otherwise $\Delta_{u}$ is set to be a positive constant such that ${\|u\|}_{1}=c_{u}$
   2. Update $v$:
      1. $v\leftarrow C^{T}u$
      2. $v\leftarrow\frac{S\left( v, \Delta_{v} \right)}{{\|S\left( v, \Delta_{v} \right)\|}_{2}}$ , where $\Delta_{v}=0$ if this results in ${\|v\|}_{1}\leq c_{v}$, otherwise $\Delta_{v}$ is set to be a positive constant such that ${\|v\|}_{1}=c_{v}$
4. If convergence is not reached after the iteration limit (default: 1000), return non-sparse weight vectors $u$ and $v$

After a weight vector pair ($h$), i.e., an LV, is found, its effect needs to be removed from the data, to look for the next possible weight vector pair ($h+1$). This process is called matrix deflation. Here, projection deflation is used as it has been shown to outperform the classic Hoteling’s deflation, which is also used in Principal Component Analysis (2,6,7). For matrices $X$ and $Y$, the deflation process from iteration $h$ to iteration$h+1$ is therefore computed as follows:

$$X_{h+1}\leftarrow X_{h}-\left( X_{h}u_{h} \right)u_{h}^{T}$$

$$Y_{h+1}\leftarrow Y_{h}-\left( Y_{h}v_{h} \right)v_{h}^{T}$$

The algorithm then uses the deflated matrices and looks for the next associative effect, i.e., the next LV. This way, SPLS iteratively provides latent variables consisting of sparse weight vector pairs ($u$, $v$), uncovering several layers of associative effects between predictor and outcome blocks.

## Machine learning framework

The models were trained and tested in a nested cross-validation framework with 10 outer ($X2$, $Y2$) and 10 inner folds ($X1$, $Y1$) (Figure S2). Individuals were randomly assigned to the fold structure. Within the inner folds, a 10x10 point grid search of both hyperparameters was conducted covering the entire hyperparameter space, in which both $l_{1}$- and $l_{2}$-norm constraints are fulfilled: $1\leq c_{u} \leq\sqrt{p} , 1\leq c_{v}\leq\sqrt{q}$ (with $p$ features in matrix$X$ and $q$ features in matrix $Y$). Lower $c_{u}$ and $c_{v}$ values lead to a sparser solution, whereas higher $c_{u}$ and $c_{v}$ values amount to a denser solution. At the upper limit, the maximum values of hyperparameters are:$c_{u}= \sqrt{p} , c_{v}= \sqrt{q}$. A SPLS analysis with $c_{u}$ and $c_{v}$ reaching these maximum values is equal to a regular PLS analysis, where every feature receives a weight and no feature is removed, i.e., no zero weights are given. Hence, our hyperparameter grid search includes the computation of one regular non-sparse PLS model (with $c_{u}$ and $c_{v}$ at the maximum limits) and an array of sparse PLS versions as lower $c_{u}$ and $c_{v}$ values are tested. Therefore, the non-sparse regular PLS solution competes against the sparse PLS solution in the hyperparameter optimization process. The weight vector pairs were generated using the training folds in the inner loop ($X1_{train}$, $Y1_{train}$):

$$\left( \boldsymbol{u,v} \right)\boldsymbol{=spls(}{\boldsymbol{X}\boldsymbol{1}}_{\boldsymbol{train}}\boldsymbol{,}{\boldsymbol{Y}\boldsymbol{1}}_{\boldsymbol{train}}\boldsymbol{,}\boldsymbol{c}_{\boldsymbol{u}}\boldsymbol{,}\boldsymbol{c}_{\boldsymbol{v}}\boldsymbol{)}$$

The model fit of the weight vector pair was then assessed by projecting them onto the testing folds ($\boldsymbol{X}\boldsymbol{1}_{\boldsymbol{test}}$, $\boldsymbol{Y}\boldsymbol{1}_{\boldsymbol{test}}$) in the inner loop and computing Spearman’s correlation coefficient between the projections of the weight vectors $\boldsymbol{u}$ and $\boldsymbol{v}$ onto their respective data matrices $\boldsymbol{X}\boldsymbol{1}_{\boldsymbol{test}}$ and $\boldsymbol{Y}\boldsymbol{1}_{\boldsymbol{test}}$:

$$\boldsymbol{\rho=|Corr}\left( \boldsymbol{X}\boldsymbol{1}_{\boldsymbol{test}}\boldsymbol{u, Y}\boldsymbol{1}_{\boldsymbol{test}}\boldsymbol{v} \right)\boldsymbol{|}$$

This approach delivers a simple and transparent measure of how well the weight vectors align the matrices to each other, i.e., how well they can maximize the covariance. The median correlation coefficient was computed for each hyperparameter combination in the inner loop. Afterwards, the best hyperparameter combinations ($\boldsymbol{c}_{\boldsymbol{u-top}}\boldsymbol{,}\boldsymbol{c}_{\boldsymbol{v-top}}$) with the highest median correlation coefficients ($\rho_{\boldsymbol{top}}\boldsymbol{)}$ were retrained on the entirety of all 10 folds of the inner loop to increase the sample size for training once more:

$$\left( \boldsymbol{u}_{\boldsymbol{top}}\boldsymbol{,}\boldsymbol{v}_{\boldsymbol{top}} \right)\boldsymbol{=spls(}{\boldsymbol{X}\boldsymbol{2}}_{\boldsymbol{train}}\boldsymbol{,}{\boldsymbol{Y}\boldsymbol{2}}_{\boldsymbol{train}}\boldsymbol{,}\boldsymbol{c}_{\boldsymbol{u-top}}\boldsymbol{,}\boldsymbol{c}_{\boldsymbol{v-top}}\boldsymbol{)}$$

The generalizability of the weight vector pairs ($\boldsymbol{u}_{\boldsymbol{top}}\boldsymbol{,}\boldsymbol{v}_{\boldsymbol{top}}$) was tested by assessing the fit of their projections onto the previously held-out fold in the outer loop and thus computing the corresponding correlation coefficients ($\rho_{\boldsymbol{max}}$).

$$\rho_{\boldsymbol{max}}\boldsymbol{=|Corr}\left( \boldsymbol{X}\boldsymbol{2}_{\boldsymbol{test}}\boldsymbol{u}_{\boldsymbol{opt}}\boldsymbol{, Y}\boldsymbol{2}_{\boldsymbol{test}}\boldsymbol{v}_{\boldsymbol{opt}} \right)\boldsymbol{|}$$

Significance testing of this weight vector pair was achieved by permutation testing against $\boldsymbol{B}$ permutations. Within the fold structure of the outer loop, $\boldsymbol{B}$ permutated datasets were created by randomly reshuffling the order of participants in one matrix ($\boldsymbol{Yb}\boldsymbol{2}$) thus destroying relationship between the two matrices. The final model with the optimized hyperparameters ($\boldsymbol{c}_{\boldsymbol{u-opt}}$, $\boldsymbol{c}_{\boldsymbol{v-opt}}$) was then retrained and tested in each of the $\boldsymbol{B}$ permuted datasets, thus generating weight vectors $\boldsymbol{u}_{\boldsymbol{b}}\boldsymbol{,}\boldsymbol{v}_{\boldsymbol{b}}$:

$$\left( \boldsymbol{u}_{\boldsymbol{b}}\boldsymbol{,}\boldsymbol{v}_{\boldsymbol{b}} \right)\boldsymbol{=spls(}{\boldsymbol{X}\boldsymbol{2}}_{\boldsymbol{train}}\boldsymbol{,}{\boldsymbol{Yb}\boldsymbol{2}}_{\boldsymbol{train}}\boldsymbol{,}\boldsymbol{c}_{\boldsymbol{u-opt}}\boldsymbol{,}\boldsymbol{c}_{\boldsymbol{v-opt}}\boldsymbol{)}$$

$$\rho_{\boldsymbol{b}}\boldsymbol{=|Corr}\left( \boldsymbol{X}\boldsymbol{2}_{\boldsymbol{test}}\boldsymbol{u}_{\boldsymbol{b}}\boldsymbol{, Y}\boldsymbol{2}_{\boldsymbol{test}}\boldsymbol{v}_{\boldsymbol{b}} \right)\boldsymbol{|}$$

Significance testing of the LV was done by assessing how often the model based on the permuted dataset performed better or equal to the model trained on the original dataset:

$$\boldsymbol{p=}\frac{\boldsymbol{1+}\sum_{\boldsymbol{b=1}}^{\boldsymbol{B}} \boldsymbol{1}_{\boldsymbol{\rho}_{\boldsymbol{b}}\boldsymbol{\geq}\boldsymbol{\rho}_{\boldsymbol{max}}}}{\boldsymbol{B+1}}$$

As our framework consisted of 10 outer folds, this approach led to 10 different models (i.e., 10 weight vector pairs $u$ and $v$) for each latent variable iteration. Of these 10 different models, we selected the one model with the best performance as measured by means of permutation testing, i.e., the model that exhibited the lowest P value. If this optimal model passed significance testing against the FDR-corrected *P* value for multiple testing (10 models resulting in a family of 10 tests), the latent variable was deemed significant and the next latent variable was computed. This concept is known as the omnibus hypothesis, which was also applied in the original method paper of the SPLS algorithm (2). The SPLS algorithm is an iterative process, in which based on hyperparameters $c_{u}$ and $c_{v}$, the weight vectors $u$ and $v$ are computed in dependence of each other. First $u$ and $v$ are initialized as non-sparse weight vectors based on regular singular value decomposition. Then an iterative process is set in motion, where first an enforcement of sparsity is attempted on weight vector $u$ in dependence of weight vector $v$. Then sparsity is enforced on $v$, based on the previously computed weight vector $u$. This iterative process is repeated, where $u$ and $v$ are sequentially updated based on each other’s previous modification until convergence between the vectors is reached. Hence, every hyperparameter setup $c_{u}$ and $c_{v}$ leads to a unique process of finding converging weight vectors $u$ and $v$ that were generated in a dialectic manner. Thus, the multivariate information is contained in this highly specific combination of weight vectors $u$ and $v$, with both vectors containing mathematical information of the other. This, in turn, makes weight vectors $u$ and $v$ from different models, such as in our 10x10 fold nested cross-validation, not suitable for usual merging techniques (weighted mean/mean/median merging or majority voting) as every vector $u$ is dependent on the corresponding vector $v$. Therefore, we used the omnibus hypothesis to determine our final LV model out of the 10 computed within the NCV structure of each LV iteration. Using our 10x10-fold outer and inner cross-validation loops can lead to high variance in the results. After training on the inner loops and then testing on the outer loops, 10 models with 10 *P* values are obtained. A criterion is then needed to determine whether any statistically significant effects were indeed found. For this, we used the omnibus hypothesis, where a statistical test is performed j-times to test a null-hypothesis H_j_. Following the omnibus approach, the combined hypothesis H_R_ over all tests j is: “All the hypothesis H_j_ are true”. This hypothesis will be rejected if any of the H_j_ hypothesis is rejected (8). In our specific case, the omnibus hypothesis states that if any of the 10 p-values (obtained in the 10 outer folds) is statistically significant (corrected for multiple testing j-times), then the omnibus hypothesis will be rejected, and the detected effect will be deemed significant. Therefore, the omnibus hypothesis will be rejected if any of the 5 splits generates a P value below .05 (adjusted for multiple testing). Of all significant splits, the model with the lowest P value will be determined as the final LV model (2). The computation ends as soon as none of the 10 splits of the LV iteration did not pass the test for significance, which renders the entire LV not significant. Since deflating the data matrices of non-significant effects would be not justified, the analysis pipeline stops after the first non-significant LV was detected.

References

1. Popovic D, Ruef A, Dwyer DB, Antonucci LA, Eder J, Sanfelici R, et al. Traces of trauma: A multivariate pattern analysis of childhood trauma, brain structure, and clinical phenotypes. Biol Psychiatry [Internet]. 2020 Dec 1;88(11):829–42. Available from: http://dx.doi.org/10.1016/j.biopsych.2020.05.020

2. Monteiro JM, Rao A, Shawe-Taylor J, Mourão-Miranda J. A multiple hold-out framework for Sparse Partial Least Squares. J Neurosci Methods [Internet]. 2016 Sept;271:182–94. Available from: http://dx.doi.org/10.1016/j.jneumeth.2016.06.011

3. Wegelin J. A survey of Partial Least Squares (PLS) methods, with emphasis on the two-block case. University of Washington, Rep T. 03/2000;

4. Witten DM, Tibshirani R, Hastie T. A penalized matrix decomposition, with applications to sparse principal components and canonical correlation analysis. Biostatistics [Internet]. 2009;10(3):515–34. Available from: http://dx.doi.org/10.1093/biostatistics/kxp008

5. Zou H, Hastie T. Regularization and variable selection via the elastic net. J R Stat Soc Series B Stat Methodol [Internet]. 2005 Apr;67(2):301–20. Available from: http://doi.wiley.com/10.1111/j.1467-9868.2005.00503.x

6. Mackey L. Deflation Methods for Sparse PCA. In Advances in Neural Information Processing Systems. 2008;(21, pp. 1017–1024).

7. Monteiro JM, Rao A, Ashburner J, Shawe-Taylor J, J. M-M. Leveraging Clinical Data to Enhance Localization of Brain Atrophy. Springer International Publishing; 2016 60–68 p. 2016;60-68p.

8. Nichols TE, Holmes AP. Nonparametric permutation tests for functional neuroimaging: a primer with examples. Hum Brain Mapp [Internet]. 2002 Jan;15(1):1–25. Available from: http://dx.doi.org/10.1002/hbm.1058
